# Bacterial vaginosis, vaginal flora patterns and vaginal hygiene practices in patients presenting with vaginal discharge syndrome in The Gambia, West Africa

**DOI:** 10.1186/1471-2334-5-12

**Published:** 2005-03-09

**Authors:** Edward Demba, Linda Morison, Maarten Schim van der Loeff, Akum A Awasana, Euphemia Gooding, Robin Bailey, Philippe Mayaud, Beryl West

**Affiliations:** 1 Medical Research Council (MRC) Laboratories, Fajara, The Gambia; 2 Department of Infectious & Tropical Diseases, London School of Hygiene & Tropical Medicine, London, UK

## Abstract

**Background:**

Bacterial vaginosis (BV) – a syndrome characterised by a shift in vaginal flora – appears to be particularly common in sub-Saharan Africa, but little is known of the pattern of vaginal flora associated with BV in Africa. We conducted a study aimed at determining the prevalence of BV and patterns of BV-associated vaginal micro-flora among women with vaginal discharge syndrome (VDS) in The Gambia, West Africa.

**Methods:**

We enrolled 227 women with VDS from a large genito-urinary medicine clinic in Fajara, The Gambia. BV was diagnosed by the Nugent's score and Amsel's clinical criteria. Vaginal swabs were collected for *T vaginalis *and vaginal flora microscopy, and for *Lactobacillus *spp, aerobic organisms, *Candida *spp and BV-associated bacteria (*Gardnerella vaginalis*, anaerobic bacteria, and *Mycoplasma *spp) cultures; and cervical swabs were collected for *N gonorrhoeae *culture and *C trachomatis *PCR. Sera were tested for HIV-1 and HIV-2 antibodies. Sexual health history including details on sexual hygiene were obtained by standardised questionnaire.

**Results:**

BV prevalence was 47.6% by Nugent's score and 30.8% by Amsel's clinical criteria. *Lactobacillus *spp were isolated in 37.8% of women, and 70% of the isolates were hydrogen-peroxide (H_2_0_2_)-producing strains. Prevalence of BV-associated bacteria were: *G vaginalis *44.4%; *Bacteroides *16.7%; *Prevotella *15.2%; *Peptostretococcus *1.5%; *Mobiluncus *0%; other anaerobes 3.1%; and *Mycoplasma hominis *21.4%. BV was positively associated with isolation of *G vaginalis *(odds-ratio [OR] 19.42, 95%CI 7.91 – 47.6) and anaerobes (P = 0.001 [OR] could not be calculated), but not with *M hominis*. BV was negatively associated with presence of *Lactobacillus *(OR 0.07, 95%CI 0.03 – 0.15), and H_2_O_2_-producing lactobacilli (OR 0.12, 95% CI 0.05 – 0.28). Presence of H_2_O_2_-producing lactobacilli was associated with significantly lower prevalence of *G vaginalis*, anaerobes and *C trachomatis*. HIV prevalence was 12.8%. Overall, there was no association between BV and HIV, and among micro-organisms associated with BV, only *Bacteroides *spp. and *Prevotella *spp. were associated with HIV. BV or vaginal flora patterns were not associated with any of the factors relating to sexual hygiene practices (vaginal douching, menstrual hygiene, female genital cutting).

**Conclusion:**

In this population, BV prevalence was higher than in corresponding populations in industrialised countries, but the pattern of vaginal micro-flora associated with BV was similar. BV or vaginal flora patterns were not associated with HIV nor with any of the vaginal hygiene characteristics.

## Background

Bacterial vaginosis (BV) is a poly-microbial syndrome characterised by a shift in vaginal flora from a predominant population of lactobacilli to their gradual or total replacement with anaerobes such as *Gardnerella vaginalis, Prevotella, Bacteroides *and *Mobiluncus *species (spp), and with other bacteria including *Mycoplasma *and *Ureaplasma *species [1]. BV is one of the most frequent conditions encountered in sexually transmitted diseases (STD), genitourinary medicine (GUM) or other reproductive health clinics throughout the world. BV has been strongly associated with poor pregnancy outcomes such as preterm delivery of low-birth-weight infants [2] and several studies have now established associations between BV and HIV [3-5]. BV appears to be particularly common in sub-Saharan Africa where several studies have reported high prevalence rates, ranging from 20–49% among women presenting to STD clinics with vaginal discharge [6-8], from 21–52% among pregnant women attending antenatal clinics [9-11], and from 37–51% in community-based studies [3,12]. These are very much higher than the rates reported from industrialised countries, 13% in GUM clinic attenders in the UK [13], 11% in gynaecology clinic attendees in London [14], and 15% to 30% in studies of non-pregnant women in USA [15].

The reasons for these disparities are not entirely clear, but may arise in part through the use of different case definitions for BV, and because the pattern of vaginal micro-flora associated with this condition may differ in different populations. Earlier African studies have relied on the Amsel's clinical definition of BV [16], whilst more recent studies have adopted the microbiological Nugent's scoring technique [17]. The latter method relies on the identification of categories of vaginal micro-flora based on quantitative assessment of a vaginal Gram-stained smear. The Nugent's method has been extensively validated in industrialised countries where numerous vaginal flora studies have been conducted [1], but little is known of the pattern of vaginal micro-flora associated with BV in Africa. The characterisation of vaginal micro-flora is an important step in understanding the pattern of flora associated with BV. This information may help investigate the significance of this condition in clinical pathology and for targeting treatment. In particular, it is important to know whether vaginal flora changes may enhance HIV acquisition as suggested [5], and to unravel some of the factors that influence such changes, as these could be perhaps modified. Behavioural factors such as vaginal douching or menstrual hygiene practices have been suggested as important factors that might influence vaginal flora composition [18], but little data is available from African populations[19,20].

We have conducted a study aiming at determining the prevalence of BV among women self-presenting with vaginal discharge at a GUM clinic in Fajara, The Gambia. We report here on the vaginal micro-flora patterns and vaginal hygiene practices found in these patients and associations with their HIV serostatus.

## Methods

### Study population and sample collection

This study was part of a WHO-sponsored evaluation of STI syndromic management and of a novel rapid diagnostic test for BV conducted at the GUM clinic of the Medical Research Council (MRC) in Fajara, The Gambia. Study details have been reported elsewhere [21]. Briefly, consecutive and consenting women aged 18 and over, attending the MRC clinic with self-reported symptoms of vaginal discharge and/or vaginal itching were included in the study. Pregnant women were excluded because it was anticipated that their vaginal flora might differ substantially from that of other women attending the clinic. A standardised questionnaire elicited socio-demographic characteristics, reproductive and sexual health history including vaginal douching and menstrual hygiene practices, and current STD symptoms. Women underwent genital examination during which vaginal and cervical swabs were collected. The first vaginal swab was used for aerobic and anaerobic cultures; the second swab was used for direct wet mount microscopy, detection of fishy amine odour ("whiff" test) when mixed with 10% potassium hydroxide (KOH) preparation, and vaginal pH determination (range 4.0–7.0); the third swab was rolled onto a slide for Gram staining; two cervical swabs were collected for *Neisseria gonorrhoeae *culture on modified Thayer-Martin media, and for *Chlamydia trachomatis *polymerase chain reaction (PCR) testing using an in-house method [22]. A blood sample was collected for HIV testing as routinely offered at the GUM clinic. Serological diagnosis of HIV infection was done according to a strategy described elsewhere [23]. In brief, sera were screened by the ICEHIV-1.O.2 (Murex Diagnostics Ltd, Dartford, UK) and reactive samples were retested by type-specific ELISAs: Wellcozyme HIV recombinant -1 (Murex) for HIV-1, and ICEHIV-2 test (Murex) for HIV-2. Samples clearly positive in one type were assigned the corresponding serological status; samples positive in both ELISAs were further tested by a synthetic peptide-based strip method, Pepti-Lav 1–2 (Sanofi Diagnostics Pasteur, Marne-La-Coquette, France).

Treatment was given to all women according to the Gambian government syndromic management protocols covering all likely vaginal and cervical infections. This included a single dose of 2.0 g of metronidazole to cover *Trichomonas vaginalis *(TV) and BV. HIV-infected patients were referred internally to our specialist clinic and managed according to local standard guidelines.

### Microbiological methods for vaginal flora assessment

The wet preparations were examined microscopically for the presence of motile TV, yeast cells, and 'clue cells'.

Vaginal smear slides were heat fixed, Gram-stained and examined for vaginal flora categories using the Nugent's method [17]. The method involves assigning a score between 0 and 10 based on quantitative assessment of the Gram-stain for three different bacterial morphotypes: (i) large Gram-positive rods (indicative of *Lactobacillus *spp), (ii) small Gram-negative or variable rods (indicative of *Gardnerella, Bacteroides *and other anaerobic bacteria), and (iii) curved, Gram-variable rods (indicative of *Mobiluncus *spp). Scores between 0 and 3 represent 'normal vaginal flora', between 4 and 6 'intermediate vaginal flora', and scores between 7 and 10 are considered diagnostic for 'BV'.

Vaginal swabs were directly inoculated at the clinic onto: (i) Columbia blood agar plates, which were incubated aerobically at 37°C for 24 to 48 hours to isolate aerobic bacteria, including lactobacilli; (ii) Columbia human blood bi-layer agar plates, which were incubated micro-aerophilically at 36°C and read after 48 to 72 hours for *Gardnerella vaginalis *isolation; (iii) Columbia-base lake horse blood kanamycin agar plates, which were incubated anaerobically at 36°C for 48 to 72 hours to isolate anaerobic bacteria; (iv) *Mycoplasma *broths incubated for 48 hrs then sub-cultured onto *Mycoplasma *agar, and incubated micro-aerophilically for 48 to 72 hours; (v) Mann Rogosa Sharpe (MRS) medium, which was used for the isolation of *Lactobacillus *spp after incubation in CO_2 _at 37°C for 48 hours; and (vi) Sabouraud's agar plates, which were incubated micro-aerophilically at 36°C 24 to 48 hour to isolate *Candida *spp.

Growth of bacterial isolates was graded as confluent (heavy growth), semi-confluent (moderate pure to mixed growth of bacteria with visible separate single colonies); and scanty (occasional single isolated colonies).

### Presumptive identification procedures

#### Lactobacilli and other aerobic flora

Lactobacilli were presumptively identified by their ability to grow well on MRS, Gram stain microscopy and catalase reaction. Isolates were further tested for their ability to produce hydrogen peroxide (H_2_O_2_) using a 2,3 tetramethyl benzedine method [24], and classified as positive when they produced blue coloration. The level of H_2_O_2 _production was determined by visually grading the intensity of the blue colour produced into low, moderate and high categories.

*Coliform *spp were identified as Gram-negative lactose fermenting rods; *Staphylococcus *spp were identified by their characteristic colony and Gram stain morphology, then tested for coagulase production (slide test); Gram-positive beta-haemolytic *Streptococcus *spp isolates were further typed using a rapid latex test according to the manufacturer's instructions (Streptex, Murex Biotech Ltd, Darford Kent, UK).

*Candida *spp were identified as colonies with typical yeast-like morphology and by characteristic morphology on a wet preparation examination (presence of budding cells and/or pseudo-hyphae).

#### BV-associated bacteria

*Gardnerella vaginalis *was identified by beta-haemolytic appearance of the colonies on human blood bilayer agar plate but not on sheep blood agars, Gram stain morphology (Gram-variable pleiomorphic coccobacilli mostly forming clumps) and negative catalase and oxidase reactions.

Suspected anaerobic isolates were sub-cultured onto Columbia blood agar without antibiotics and incubated in aerobic and anaerobic conditions. Strict anaerobes were further identified by Gram staining and antibiotic susceptibility to erythromycin, rifampicin, colistin, penicillin, kanamycin and vancomycin (Oxoid Discs, Unipath, Basinstoke, Hampshire UK). Gram-negative anaerobic bacilli were tested and the isolates which grew on bile medium and hydrolysed aesculin were identified as *Bacteroides *spp. Isolates which failed to grow on bile or hydrolyse aesculin were identified as *Prevotella *spp [25].

*Mycoplasma *spp were identified as typical "fried egg" colonies and stained with Diene's stain. They were further presumptively identified as *Mycoplasma hominis *by colonial appearance and staining characteristics with a permanent diffused light blue periphery and a darkly blue centre [25].

### Diagnosis of bacterial vaginosis

The gold standard microbiological definition of BV was a score of 7–10 by the Nugent's method described above. Amsel's clinical criteria were also used to make a clinical diagnosis of BV, which included the presence of any three of the following; (i) homogeneous grey adherent vaginal discharge; (ii) vaginal fluid pH ≥ 4.6; (iii) release of fishy amine odour when 10% potassium hydroxide (KOH) solution was added to a sample of vaginal fluid (the "whiff" test); and (iv) the presence of 'clue cells' representing over 20% of vaginal epithelial cells on wet-prep microscopic examination [16].

Ten percent random quality control checks were carried out on the microscopist by an experienced BV microscopist, for Nugent's score and wet preparation readings for 'clue cells'.

### Statistical methods

Frequency tables were produced to describe the prevalence of genital infections and of micro-flora isolates. *Bacteroides, Prevotella, Peptostreptococcus *and other anaerobic isolates were grouped as 'anaerobes'. Chi-square tests were used to examine the association between isolates and BV diagnosis by Nugent's score and Amsel's clinical criteria in univariate analyses. Logistic regression was used to estimate the association between each isolate and diagnosis of BV by one method adjusted for (i.e. within categories of) BV diagnosis by the other method; and to test whether the observed associations were different between categories of the other method (i.e. testing for interaction). Chi-square and Fisher's Exact (for small numbers) tests were used to examine associations between categorical variables such as: isolation of lactobacilli, particularly H_2_O_2_-producing strains and *G vaginalis*, anaerobic isolates, *Mycoplasma hominis*, as well as *N gonorrhoeae, C trachomatis, Candida *spp or *T vaginalis; *HIV and each of the vaginal flora micro-organisms mentioned above, and with BV (Nugent's score 7–10); HIV and vaginal hygiene variables; and between BV and vaginal hygiene variables.

### Ethical issues

The study was approved by the Ethical Committees of the MRC Gambia and the London School of Hygiene & Tropical Medicine, and by the Ethics Review Board of the World Health Organisation.

## Results

Two hundred and thirty women were enrolled in the study over a four-month period in 2000, but data of three women were subsequently excluded because the women had not reported any symptoms consistent with our case definition of vaginal discharge syndrome. The denominators used in subsequent analyses vary slightly depending on completeness of microbiological investigations.

The median age of the patients was 26 years (range 18–50), 71% of women were married, and a majority of women (84%) reported only one sexual partner in the last three months. Antibiotic use prior to attending the clinic was reported by 18% of patients.

### Clinical and aetiological findings

The prevalence of cervical infections was 6.3% (14/222) for *N gonorrhoeae *and 15.0% (34/227) for *C trachomatis*. A serum sample was obtained from 210 (93%) women and 27/210 (12.8%) of them were HIV infected (19 with HIV-1, 7 with HIV-2, and 1 dually with HIV1 and HIV-2).

Using wet preparation microscopy, 10.1% (23/227) of the women were positive for *T vaginali*s and 22.9% (52/227) for yeast cells, with only two patients having a dual infection. 'Clue cells' were seen in 56.4% of the wet preparations but in only 23.1% of the Gram-stained smears. A large majority of women (91.2%) had an elevated pH (≥ 4.6). Overall, 30.8% (68/221) of women had a diagnosis for BV according to the Amsel's criteria.

BV prevalence as determined by Nugent's score of 7–10 was 47.6% (108/227), 24.7% (56/227) women had 'intermediate flora' (score 4–6) and 27.7% (63/227) had 'normal flora' (score 0–3).

### Vaginal flora cultures

Results of vaginal flora cultures for aerobic, micro-aerophilic and anaerobic bacteria as well as cultures for *Mycoplasma *are shown in Table 1. Variations in the denominators for the types of bacteria isolated were due to cases of either contamination or growth failure. The lower number of anaerobic cultures performed (n = 66) was due to a breakdown in the supply of anaerobic Gas-Packs. We defined two unidentified Gram-positive anaerobic isolates as 'Other' anaerobic isolates.

**Table 1:** Vaginal flora isolates by Nugent's score among women presenting with vaginal discharge syndrome at the GUM clinic in Fajara, The Gambia

	**Total Samples**		**Scanty Growth**		**Semi-confluent growth**		**Confluent growth**		***P-value^2^***
**Nugent's score^1^**	**0–6**	**7–10**	**0–6**	**7–10**	**0–6**	**7–10**	**0–6**	**7–10**	
**Isolate**	**N**	**N**	**% (n)**	**% (n)**	**% (n)**	**% (n)**	**% (n)**	**% (n)**	

**Aerobic flora**									
*Lactobacillus *spp.	119	106	5.0 (6)	8.5 (9)	16.8 (20)	4.7 (5)	33.6 (40)	4.7 (5)	*<0.001*
*Coliform *spp.	119	106	12.6 (15)	9.4 (10)	1.7 (2)	6.6 (7)	5.0 (6)	4.7 (5)	*0.868*
*Staphylococcus *spp.	119	105	24.4 (29)	37.1 (39)	9.24 (11)	4.8 (5)	0.8 (1)	0.9 (1)	*0.217*
*Streptococcus *spp.	119	105	13.4 (16)	15.2 (16)	11.8 (14)	12.4 (13)	13.4 (16)	10.9 (11)	*1.000*
*Candida *spp.	93	81	11.8 (11)	14.8 (12)	16.1 (15)	7.4 (6)	15.1 (14)	8.6 (7)	*0.117*
**Microaerophilic flora**									
*Gardnerella vaginalis*	117	106	1.7 (2)	8.5 (9)	6.8 (8)	21.7 (23)	13.7 (16)	38.7 (41)	*<0.001*
**Anaerobic flora**									
Any anaerobic isolate^3^	34	32	1 (3)	2 (6)	2 (6)	9 (28)	3 (9)	6 (19)	*0.005^4^*
*Bacteroides *spp.	34	32	0	6.2 (2)	0	12.5 (4)	2.9 (1)	12.5 (4)	*0.002*
*Prevotella *spp.	34	32	2.9 (1)	0	2.9 (1)	9.4 (3)	2.9 (1)	12.5 (4)	*0.180*
*Peptostreptococcus *spp.	34	32	0	0	0	3.1 (1)	0	0	*0.485*
Other anaerobes^5^	34	32	0	0	1 (3)	2 (6)	1 (3)	0	*0.801*
*Mobiluncus *spp.	35	33	0	0	0	0	0	0	-
*Mycoplasma hominis*	115	105	0	0	2.6 (3)	0.9 (1)	14.8 (17)	24.8 (26)	*0.142*

Note: denominators vary according to the number of samples for which each test was conducted (see Results section).

^1 ^Nugent's categories: score 0–6 = normal and intermediate flora; score 7–10 = BV.

^2 ^From Fisher's Exact test.

^3 ^Includes *Bacteroides *spp., *Prevotella *spp. and *Peptostreptococcus *spp. isolates and other anaerobes.

^4 ^Anaerobic isolates were found in 21 samples out of 66; 5 women had more than 1 anaerobic isolate in which case the highest level of growth was used for the significance test.

^5 ^Anaerobic Gram-negative rods, anaerobic Gram-positive or pigmented anaerobes.

*Gardnerella vaginalis*, lactobacilli, streptococci and *Mycoplasma *spp were present as confluent or semi-confluent growth. *Mobiluncus *spp were not isolated. *Staphylococcus *spp, and *Coliforms *spp were mostly present in scanty numbers. *Bacteroides *and *Prevotella *spp were present in semi-confluent to confluent numbers.

Seventy percent (42/60) of lactobacilli tested were found to be H_2_O_2_-producers, 12 at high level, 12 at intermediate level, and 18 at low levels of H_2_O_2 _production. We could not establish the ability of the remaining 25 lactobacilli isolates to produce H_2_O_2 _because these isolates could not be recovered from storage.

*Staphylococcus *spp were tested for coagulase production and 20.5% (17/83) were identified as *Staphylococcus aureus*. Serotype grouping was done for 61/86 *Streptococcus *spp isolates which were successfully recovered and the majority of these, 68.9% or 42 strains, were classified as Group B, 2 as group C, 6 as group D, and 8 as group F.

### Associations between bacterial vaginosis and vaginal flora

**Figure 1:**
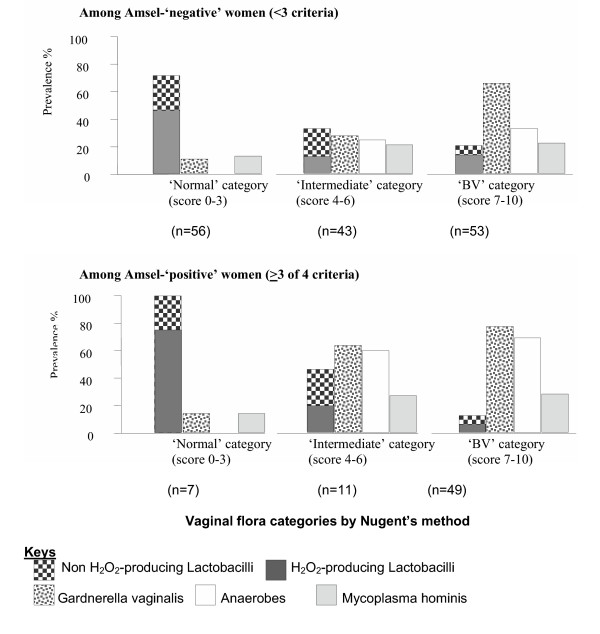
Prevalence of microflora isolates by Amsel's clinical criteria diagnosis and Nugent's vaginal flora categories

Patterns of micro-flora isolates according to BV diagnosis by Nugent's score and Amsel's clinical criteria are summarised in Table 2 and Figure 1. Isolation of lactobacilli and H_2_O_2_-producing lactobacilli was negatively associated with BV diagnosis by both Nugent's score (OR 0.07, 95%CI 0.03 – 0.15) and Amsel's criteria (OR 0.49, 95%CI 0.26 – 0.92). Within Amsel's diagnostic categories, the Nugent's diagnosis of BV was still negatively associated with lactobacilli (adjusted OR [AOR] 0.06, 95%CI 0.03 – 0.15). However, within Nugent's diagnostic categories, Amsel's diagnosis of BV was not associated with lactobacilli (AOR 1.11, 95%CI 0.52 – 2.38). *G vaginalis *and the anaerobes were strongly associated with BV diagnosis by both Nugent's score and Amsel's criteria. *G vaginalis *remained significantly associated with BV diagnosis by either method, within the diagnosis categories of the other method. It was not possible to compare the prevalence of anaerobes across the three Nugent categories within Amsel's diagnosis using a likelihood ratio test due to the lack of anaerobic isolation in normal subjects. *M hominis *was more common in women with BV by both Nugent's and Amsel's criteria but the association was not statistically significant. *Coliform *spp, staphylococci and streptococci were not significantly associated with BV by either Nugent's score or Amsel's criteria (data not shown).

**Table 2:** Association between vaginal micro-flora isolates and Nugent's vaginal flora categories and Amsel's diagnosis of bacterial vaginosis

		**Association with Nugent's vaginal flora categories**				**Association with Amsel's diagnosis of BV**				
	
**Isolate**	**Nugent^1^**	**N^2^**	**%^3^**	**OR^4 ^95% CI**	**AOR^5 ^95% CI**	**Amsel^6^**	**N^2^**	**%^3^**	**OR^4 ^95% CI**	**AOR^5 ^95% CI**
**Lactobacilli**	Normal flora	63	74.6	1	1					
	Interm.	54	35.2	0.18 0.08–0.41	0.18 0.08–0.41	Neg.	152	42.8	1	1
	BV	102	16.7	0.07 0.03–0.15	0.06 0.03–0.15	Pos.	67	26.9	0.49 0.26–0.92	1.11 0.52–2.38
	*P^7^*			*<0.001*	*<0.001*	*P^7^*			*0.025*	*0.775*
**H_2_O_2 _- Lactobacilli**	Normal flora	49	49.0	1	1					
	Interm.	49	14.3	0.17 0.07–0.46	0.17 0.07–0.47	Neg.	134	24.6	1	1
	BV	97	10.3	0.12 0.05–0.28	0.12 0.05–0.31	Pos.	61	13.1	0.46 0.20–1.07	0.94 0.36–2.49
	*P^7^*			*<0.001*	*<0.001*	*P^7^*			*0.067*	*0.909*
***G vaginalis***	Normal flora	61	11.5	1	1					
	Interm.	54	35.2	4.19 1.59–11.0	3.96 1.50–10.5	Neg.	150	35.3	1	1
	BV	102	71.6	19.42 7.91–47.6	15.45 6.18–38.6	Pos.	67	68.7	4.01 2.17–7.42	2.21 1.10–4.44
	*P^7^*			*<0.001*	*<0.001*	*P^7^*			*<0.001*	*0.026*
**Anaerobic isolates^8^**	Normal flora	21	0	1	1					
	Interm.	13	38.5	-^9^	-^9^	Neg.	43	18.6	1	1
	BV	31	48.4	-^9^	-^9^	Pos.	22	54.6	5.25 1.68–16.38	4.5 1.24–16.37
	*P^7^*			*0.001*	*-^10^*	*P^7^*			*0.003*	*0.018*
***Mycoplasma hominis***	Normal flora	60	13.3	1	1					
	Interm.	53	22.6	1.90 0.71–5.09	1.85 0.69–4.97	Neg.	148	18.9	1	1
	BV	102	25.5	2.22 0.93–5.29	2.00 0.80–4.96	Pos.	67	26.9	1.57 0.80–3.10	1.34 0.65–2.76
	*P^7^*			*0.184*	*0.282*	*P^7^*			*0.188*	*0.436*

^1 ^Nugent's categories: normal flora (score 0–3), intermediate flora (score 4–6), BV (score 7–10).

^2 ^Number of specimens.

^3 ^Percentage positive for the isolate.

^4 ^Crude Odds Ratio.

^5 ^Odds Ratio adjusting for the other method of diagnosing BV.

^6 ^Amsel's diagnosis 'positive' [pos.] if at least 3 out of 4 criteria present (homogenous adherent discharge; pH ≥ 4.6; whiff test positive; clue cells on 20% of epithelial cells); diagnosis is 'negative' [neg.] if <3 criteria present.

^7 ^From *X*^2 ^test for unadjusted analysis. From likelihood ratio test when adjusting for the other diagnostic method.

^8 ^Anaerobic isolates comprise *Prevotella *spp, *Bacteroides *spp, *Peptostreptococcus *spp and other anaerobes.

^9 ^Not possible to calculate OR relative to normal flora due to lack of anaerobic isolation in normal subjects.

^10 ^Not possible to fit model due to lack of anaerobic isolation in normal subjects.

The associations between the presence of lactobacilli, including the presence of H_2_O_2 _producing lactobacilli strains, and the isolation of other vaginal or cervical organisms are shown in Table 3. There were significant 2 to 4 fold decreases in colonisation by *G vaginalis *or anaerobes in the presence of H_2_O_2_-producing lactobacilli (P < 0.001 and P = 0.016 respectively). *M hominis *isolation was less prevalent in the presence of H_2_O_2_-producing lactobacilli, but this was not statistically significant. Overall there was no significant difference for *Candida *isolates (P = 0.108) but the lowest prevalence was observed when no lactobacilli were isolated. Detection of *C trachomatis *was significantly reduced (p = 0.024) in the presence of H_2_O_2_-producing lactobacilli strains, whilst *N gonorrhoeae *was isolated more frequently in the absence of lactobacilli, although this was not statistically significant.

**Table 3:** Association between lactobacilli and other genital micro-organisms

**Genital micro-organisms**	**Prevalence of genital micro-organism in those with:**						
	
	**No lactobacilli **		**Non H_2_O_2 _producing lactobacilli**		**H_2_O_2 _producing lactobacilli**		***P-value^1^***
	**n/N**	**(%)**	**n/N**	**(%)**	**n/N**	**(%)**	
*C trachomatis*	21/140	(15.0)	4/18	(22.2)	1/42	(2.4)	*0.024*
*N gonorrhoeae*	12/138	(8.7)	0/17	(0)	1/42	(2.4)	*0.303*
*Candida *spp	34/110	(30.9)	6/14	(42.9)	16/32	(50.0)	*0.108*
*T vaginalis*	19/140	(13.6)	1/18	(5.6)	3/39	(7.1)	*0.509*
*G vaginalis*	78/140	(55.7)	5/18	(27.8)	9/40	(22.5)	*<0.001*
Anaerobes	16/34	(47.1)	0/3	(0)	3/21	(14.3)	*0.016*
*M hominis*	33/137	(24.1)	5/17	(29.4)	5/40	(12.5)	*0.214*

^1 ^From Fisher's Exact test because of small numbers in some categories.

### Associations between vaginal flora, vaginal hygiene practices and HIV

We found that only two anaerobic isolates, *Bacteroides *spp and *Prevotella *spp, were significantly more common among HIV positive women: 7/57 samples (12.3%) for HIV negative women contained *Bacteroides *spp, compared with 3/6 (50%) for HIV-positive women (P = 0.0046); and 6/57 samples (10.5%) for HIV negative women contained *Prevotella *spp, compared with 3/6 (50%) for HIV positive women (P = 0.033). In overall crude analysis, there was no association between BV (Nugent's score 7–10) and HIV (all types): 12/110 (10.9%) women without BV were HIV-infected vs. 15/100 (15.0%) women with BV (table 4).

**Table 4:** Prevalence of HIV and BV in symptomatic women, by douching, menstrual hygiene protection method and female genital cutting

	**Prevalence of BV (Nugent's score 7–10)**			** Prevalence of HIV **		
	
	**n/N**	**%**	***P-value^4^***	**n/N**	**%**	***P-value^4^***
**Douching^1^**						

No	12/22	54.5	*0.507*	3/22	13.6	*1.000*
Yes	94/202	46.5		24/185	13.0	

**Menstrual hygiene protection method**						

Traditional^2^	70/138	50.7	*0.323*	17/129	13.2	*1.000*
Sanitary pads	34/79	43.0		9/71	12.7	

**Female genital cutting^3^**						

No	38/68	55.9	*0.146*	12/63	19.1	*0.115*
Yes	70/157	44.6		15/145	10.3	

**BV**						

No				12/110	10.9	*0.414*
Yes				15/100	15.0	

^1 ^Douching before and/or after sexual intercourse.

^2 ^Old cloths washed and re-used as necessary.

^3 ^Also known as "female circumcision"; in the Gambia, the type of female cutting belongs to WHO classification Type II, i.e. removal of all/part of the clitoris and labia minora.

^4 ^P-values from Fisher's Exact Test.

Details about vaginal hygiene practices were collected by questionnaire: 37.8% (85/225) of women reported practicing vaginal washing before sex and 89.2% (199/223) after sex, for a combined 90.2% of women (202/224) practising some form of 'douching' before or after sex. Of the women who douched, 57.6% (114/198) used water, 22.2% (44/198) used soap and water, 18.2% (36/198) used a dry towel, and 2% (4/198) used commercial or other cosmetic products. The source of water was 83% tap water, and 17% water from a protected well. In crude univariate analysis, there was no association between 'douching' and BV (Nugent's score 7–10) (Table 4), nor between any of the individual vaginal hygiene variables and BV (data not shown). The questionnaire also explored forms of menstrual hygiene: 60.8% (118) of women reported using traditional methods for sanitary protection (i.e. reusable cloths), 34.8% (79) using sanitary pads, 4.0% (9) tampons and 0.4% (1) other methods. Again, there were no associations between these methods and presence of BV (table 4). The majority of women (157/227, 69.2%) were found circumcised on examination. The dominant form of genital cutting in the Gambia belonged to WHO classification Type II (i.e. removal of all/part of the clitoris and labia minora) as reported previously [26]. Again no association with BV was noted (table 4). In overall crude univariate analysis, we did not find any association between douching, menstrual hygiene, genital cutting and HIV (table 4).

## Discussion

The main objective of this study was to determine the prevalence of BV and the pattern of vaginal micro-flora among women with vaginal discharge syndrome in an African setting and to relate this to vaginal hygiene practices and HIV serostatus.

Using Nugent's score as the gold standard, a BV prevalence of 47.6% was found in this population. This compares to the range (20–49%) reported from other African populations attending STI clinics: 20–23% in Burkina Faso [7] and Malawi [27], 37% in Tanzania [8] and 49% in Kenya [6]. High prevalence of BV (21–29%) has also been observed among pregnant women in Kenya and South Africa [9,11,28], The reasons for higher BV rates in African populations are not known. High BV rates have been reported among African-American women [15], although it has been argued that levels of education and other socio-economic factors were confounding these associations [15,29]. Lifestyle practices such as vaginal douching have also been associated with an increased prevalence of BV [15,18,19], although, the direction of causality is again uncertain, since most studies have been of cross-sectional nature and many potentially confounding factors such as educational, socio-economical and behavioural factors have not always been entirely controlled for. We did not find any association between BV or vaginal micro-organisms and vaginal hygiene practices such as douching before or after sex, the nature of douching compounds used, the source of the water, or with menstrual sanitary protection. This finding perhaps owes to the fact that a very large proportion of women reported these practices, thus any relatively small association with BV would be hard to find with our sample size. On the other hand, additional possible explanations for the high prevalence or incidence of BV in African populations have to be sought – the role of hormonal factors should be explored. An association between BV and HIV has been reported in several studies [3,4,30], possibly influenced by vaginal hygiene practices [18,20,31]. However, as in our study, not all studies reporting on douching, BV and HIV have found associations between these factors [19]. The relationship between HIV, risk for BV or other STIs is complex, and could be contributed to by high risk sexual behaviour. Our study population consisted only of symptomatic women attending a GUM clinic, thus high-risk behaviours may have blurred any possible association. To our knowledge, this study is one of the first to report on female genital cutting in relation to HIV and vaginal flora in Africa. We did not find any significant impact of circumcision on vaginal flora or HIV serostatus.

A comparison of vaginal micro-flora isolates with Nugent's score showed significant positive associations between a diagnosis of BV and the isolation of *G vaginalis*, a significant positive association with anaerobes, and a significant negative association with the presence of lactobacilli. These findings are not surprising, since the method employed for the Nugent's score is based on the observation of BV-associated bacterial morphotypes. Nonetheless there have been no reports of vaginal micro flora culture studies in African population aimed at exploring the patterns of BV-associated flora. This study has demonstrated similar vaginal bacterial isolates to those found in the United States [32,33]. These studies found a strong association between BV and the isolation of *G vaginalis*, anaerobic gram-negative rods belonging to the genera *Prevotella, Porphyromonas *and *Bacteroides*, *Peptostreptococcus *spp, *M hominis*, *Ureaplasma urealyticum*, and often *Mobiluncus *spp.

A lower concentration of facultative species of *Lactobacillus *among women with BV in comparison to women with a normal flora was noted in this study. Lactobacilli are reported to play an important role in the maintenance of normal vaginal flora [34,35] through the provision of defence mechanisms against pathogenic organisms via hydrogen peroxide (H_2_0_2_) production and the maintenance of an acidic microenvironment generated by lactic acid production. A low pH has been shown to have a direct microbicidal and virucidal effect [36]. Hydrogen peroxide, which is produced by some lactobacilli strains, also has a direct antimicrobial effect. H_2_O_2 _can inhibit the growth of *Bacteroides, Gardnerella, Mobiluncus*, *Mycoplasma *and other vaginal organisms by acting directly on these organisms using its toxic effect, or by reacting with halide ions in the presence of vaginal peroxidase as part of the H_2_O_2_-halide peroxidase antibacterial system. Lactobacilli can also adhere onto vaginal epithelial cells [37] thus blocking the attachment of any pathogenic BV-associated bacteria onto these cells, Lactobacilli are known to produce biosurfactant, bacteriocins and coaggregation molecules [38], all of which contribute to the maintenance of a healthy vaginal micro-environment.

In our study, a large proportion of lactobacilli isolates (70%) were H_2_O_2 _producers. These isolates were associated with a significantly lower prevalence of *G vaginalis*, anaerobes and *C trachomatis*, with trends of lower prevalence of *N gonorrhoeae *and *M hominis*, suggesting a protective effect of vaginal/cervical colonisation conferred by these lactobacilli strains. The absence of H_2_0_2_-producing lactobacilli was not associated with growth of *Candida *spp. This is different from the findings observed in a study conducted by Hillier *et al *[35], in which significant associations between H_2_O_2 _production and protection against BV and other STI as well as protection against symptomatic candidiasis were reported.

In previous studies [39,40], *M hominis *and *Ureaplasma urealyticum *have been associated with BV, although not in all cases [41]. In our study, there appears to be some association between *M hominis *and BV, defined either by Nugent's score or Amsel's criteria, but these were not statistically significant. This observed difference may be due to the different detection protocols employed by different researchers.

Other bacteria that have been isolated in this study included *Staphylococcus *spp (38.4%), *Coliform *spp (20.0%)and *Streptococcus *spp (38.4%) but they were not associated with any particular vaginal microflora. Most of the staphylococci isolates were coagulase-negative, which are perceived to be normal commensal organisms. Most streptococci were found to belong to group B, an organism which can be highly pathogenic, particularly at time of delivery when it has been associated with neonatal sepsis and premature delivery [42]. The high prevalence of Group B streptococci seen in this study was similar to that reported in previous work done in The Gambia [43]. The latter study, however, failed to demonstrate any association between isolation of Group B streptococci and disease manifestation in neonates. Thus, the high prevalence of group B streptococci isolates reported in this study may not pose any significant clinical problem in this setting.

Several logistical problems have arisen in the course of this study, which may limit interpretation of the data on vaginal flora patterns. First, *Mobiluncus *spp, were not isolated despite observing *Mobiluncus*-like organisms in some of the vaginal Gram-stained smears. This finding could be attributed to a low prevalence of *Mobiluncus *in our population, or to an inadequate isolation procedure, which will warrant further investigation. Second, our adoption of a 48-hours incubation period for broth- and agar- *Mycoplasma *culture procedures did not favour the isolation of *Ureaplasma urealyticum*, as the latter is sensitive to the metabolic by-products generated during extended incubation periods. This may have been partly responsible for the lack of *Ureaplasma *isolation in this study. Finally, there was a low-recovery rate for a significant number of stored isolates. Optimum storage procedures are not easy to maintain in tropical climates. These logistical problems with fastidious organisms may explain why it is difficult to attempt studies of vaginal flora in developing countries.

Another possible limitation of our study was the absence of inclusion of women who did not complain of vaginal discharge syndrome, which would have allowed a more comprehensive description of patterns of vaginal flora in symptomatic and symptomless women. Our study shows, however, that the pattern of organisms cultured in BV in this environment is similar to what has been reported in corresponding populations in industrialised countries, and therefore suggest that different vaginal flora patterns are not the major explanation behind the higher prevalence of BV in Africa.

In most STI or GUM clinics in industrialised countries, routine BV diagnosis is made using Nugent's score or Amsel's clinical criteria. In resource-poor settings good microscopy is not often available, and clinicians rely on clinical judgement or may apply all or part of the Amsel's clinical criteria. This study found there was an association between Amsel's criteria and Nugent's score, a strong correlation between Amsel's diagnosis and the presence of *G vaginalis *and anaerobes, and a negative correlation with the presence of lactobacilli. However, as shown in Figure 1, a large number of true BV cases (by Nugent's score) were missed by the Amsel's method, limiting its utility as a BV diagnostic method. The Amsel's method can be highly subjective with regards the description of the discharge and the olfactive component ('whiff' test); the wet mount microscopy depends on the experience of the microscopist and can represent a further subjective element, particularly when performed under pressing clinical conditions [1]. Despite regular supervision and the use of the same clinician and microscopist throughout the study (which insured internal consistency of our results), the discrepancies we found between the Amsel's criteria and the accepted gold standard diagnosis (Nugent's method) in this study appear to preclude the use of this method under routine clinical conditions in our setting.

Our study found that where Gram-staining led to a classification of 'intermediate flora' by Nugent's score, this was reflected in the microbiological findings, which were 'intermediate' quantitatively and qualitatively between 'normal' and 'BV' categories (table 2 and figure 1) and distinct from them. This supports the validity of the classification and could indicate that the 'intermediate' flora precedes the development or follow the resolution of frank BV. Longitudinal studies are required to elucidate this phenomenon.

## Conclusion

In this population, BV prevalence was higher than in corresponding populations in industrialised countries, but the pattern of vaginal micro-flora associated with BV was similar. BV or vaginal flora patterns were not associated with HIV nor with any of the vaginal hygiene characteristics. Further studies on the public health significance of BV in this kind of setting are needed to determine future strategies for intervention

## Competing interests

We received donation of FemExam^® ^tests from Litmus Ltd (CA, USA) for this study.

## Authors' contributions

ED contributed to study design, conducted all laboratory investigations and produced the first draft of the manuscript; LM oversaw data management and conducted statistical analyses; MSL oversaw clinical data collection and provided epidemiological support; AAA contributed to clinical data management; EG conducted the clinical part of the study; PM, RB and BW developed the study protocol and supervised the study with BW overseeing the laboratory aspects of the study; all authors contributed revisions to the manuscripts.

## Pre-publication history

The pre-publication history for this paper can be accessed here:


